# Neighbour noise annoyance is associated with various mental and physical health symptoms: results from a nationwide study among individuals living in multi-storey housing

**DOI:** 10.1186/s12889-019-7893-8

**Published:** 2019-11-12

**Authors:** Heidi A. R. Jensen, Birgit Rasmussen, Ola Ekholm

**Affiliations:** 1 National Institute of Public Health, University of Southern Denmark, Studiestræde 6, 1455 Copenhagen, Denmark; 2 Danish Building Research Institute, Aalborg University, A.C. Meyers Vænge 15, 2450 Copenhagen, Denmark

**Keywords:** Noise, Noise annoyance, Neighbour noise, Pain, Headache, Fatigue, Depression, Health surveys

## Abstract

**Background:**

Noise exposure is considered a stressor that may potentially exert negative health effects among the exposed individuals. On a population basis, the most prevalent and immediate response to noise is annoyance, which is an individually experienced phenomenon that may activate physiological stress-responses and result in both physical and mental symptoms. Health implications of traffic noise have been investigated thoroughly, but not of neighbour noise. The aim of the present study was to examine the associations between neighbour noise annoyance and eight different physical and mental health symptoms.

**Methods:**

Cross-sectional data from the Danish Health and Morbidity Survey 2017 were used. The present study included a random sample of 3893 adults living in multi-storey housing. Information on neighbour noise annoyance and various health symptoms (e.g. pain in various body parts, headache, sleeping problems, depression, and anxiety) during the past two weeks was obtained by self-administered questionnaires. The question on neighbour noise annoyance and health symptoms, respectively, had three possible response options: ‘Yes, very annoyed/bothered’, ‘Yes, slightly annoyed/bothered’, ‘No’. The associations between neighbour noise annoyance and very bothering physical and mental health symptoms were investigated using multiple logistic regression models.

**Results:**

Being very annoyed by neighbour noise was significantly associated with higher odds of being very bothered by all eight health symptoms (adjusted OR = 1.73–3.32, all *p*-values < 0.05) compared to individuals not annoyed by noise from neighbours. Statistically significant interactions were observed between sex and two of the eight health symptoms. Among women, a strong association was observed between neighbour noise annoyance and being very bothered by pain or discomfort in the shoulder or neck, and in the arms, hands, legs, knees, hips or joints. Among men, no associations were observed.

**Conclusions:**

Based on the findings from this study, neighbour noise annoyance is strongly associated with eight different physical and mental health symptoms. Future studies are encouraged to 1) determine the direction of causality using a longitudinal design, 2) explore the biological mechanisms explaining the sex-specific impact of neighbour noise annoyance on symptoms of musculoskeletal pain or discomfort and the other outcomes as well.

## Background

Noise exposure is considered a stressor that may potentially exert negative health effects among the exposed individuals. The negative impact of environmental noise on health is increasingly being recognised worldwide [1–4]. The estimated number of disability-adjusted life years (DALYs) lost because of environmental traffic noise is 45,000 years for cognitive impairment in children, 61,000 years for ischemic heart disease, 654,000 years for annoyance and 903,000 years for sleep disturbances in Western European countries [5]. Corresponding DALYs lost due to neighbour noise are, unfortunately, not available. Annoyance from both traffic noise and neighbour noises are dealt with in detail in [2, 3], but the methodology is quite different from [1, 4], and health implications are not counted. The WHO Noise Guidelines from 2018 includes revised recommendations for noise limits for road traffic, railways and air traffic, but – in addition to transportation noise as before – it includes also recommendations for wind turbine noise and leisure noise [4]. However, neighbour noise is not dealt with in the guidelines. Not even in the review papers prepared as basis for the guidelines.

On a population basis, the most prevalent and immediate response to environmental noise is annoyance [6, 7]. Traffic noise is a physically measurable stimulus, but also an individually experienced phenomenon, which is reflected in the degree of neighbour noise annoyance [8]. Noise from neighbours could be enjoyed and appreciated by some individuals like e.g. people’s laughters and birds chirping, but barking dogs, children crying or screaming, neighbours arguing, or even domestic violence may result in an adverse reaction [9]. Even though noise annoyance is not considered a direct health outcome itself, it may modify the causal pathway between noise and health by inducing negative emotional reactions such as irritability, distress or other stress-related symptoms in the affected individuals [10]. Eventually, such reactions may be translated into physiological reactions by an activation of both ‘direct’ and ‘indirect’ pathways of stress reactions, sleep-stage changes and other biological and biophysical effects [11]. This may in turn negatively affect various health risk factors such as blood pressure, circulating blood lipids and glucose levels [12] and heartbeat frequency and induce changes in the release of the activation hormones adrenalin, noradrenalin and cortisol in body fluids [13]. In this way, the regulation of vital body functions can be compromised, which may increase the risk of common non-communicable diseases such as e.g. cardiovascular diseases [12], respiratory diseases [14], and metabolic diseases [15].

While for traffic noise, there is a clear relationship between objectively estimated noise exposure and noise annoyance [16], several non-acoustic factors affect noise annoyance, of which noise sensitivity is considered one of the most important [10, 17]. In a population-based study, Park and colleagues [18] even found noise sensitivity, rather than traffic noise level, to predict negative non-auditory effects of noise such as depression, anxiety and insomnia. Noise sensitivity is regarded as a stable personality trait that reflects an individual’s attitude towards noise in general [19] and is affected by complicated interactions between e.g. stressors and coping strategies developed by a subject through e.g. previous experiences, psychological, biological and social factors [20]. Noise sensitivity is believed to moderate the degree of noise annoyance, and thus partly explain the inter-individual variance of reactions to the same level of noise [21–24]. However, over time personal feelings, reactions and attitude may change based on the personal history of noise events and how the related noise sources are addressed in the society.

In previous research investigating the negative impact of environmental noise and noise annoyance on health, the focus has mainly been on aircraft noise, railway noise, road traffic noise and (during the latest decades) also wind turbine noise, which can be objectively quantified by physical parameters [25]. However, there seems to be a growing awareness in other types of noise sources, such as neighbour noise, which has a relatively high annoyance potential because of, for example, the unpredictable nature and the high information content (e.g. in speech, music and footsteps) [8]. Epidemiological studies have found neighbour noise annoyance to be negatively associated with indicators of both physical [10, 13, 26] and mental health [7, 10, 13, 26, 27]. Moreover, a previous Danish study found a dose-response relationship between the degree of neighbour noise annoyance and poor mental health and high levels of perceived stress, respectively [28]. Interestingly, a German study compared different sources of noise annoyance in order to assess their impact on mental health and found neighbour noise annoyance to have a more negative impact on mental health than both traffic and aircraft noise annoyance [27].

Thus, there are indications that the distinct nature of neighbour noise annoyance may potentially exert harmful effects on human health to an extent that remains to be fully elucidated. Moreover, previous studies have suggested potential sex-specific associations between noise annoyance and health [e.g. 29–32]. These studies, however, showed somewhat conflicting results on this matter, which calls for a clarification of such potential sex-specific associations.

As modern society has developed towards a ‘loud’ society with noise stimuli surrounding us nearly 24 h a day [13], it is of the utmost importance to expand the current knowledge on the association between understudied noise annoyance sources, including neighbours, and health. Thus, the aim of the present study was to examine the association between neighbour noise annoyance and different physical and mental health symptoms among individuals living in multi-storey housing. A secondary aim was also to explore whether there are sex-specific effects embedded in the potential association between noise annoyance and health symptoms.

## Methods

In the present study, we use cross-sectional data from the Danish Health and Morbidity Survey in 2017 [33]. A nationally representative random sample of 25,000 adults (16 years or older) was drawn from the Danish Civil Registration System [34].

Initially, an introductory letter was sent to all selected individuals in the sample that briefly described the purpose and content of the survey. Participation in the survey was voluntary. Data were collected using a self-administered questionnaire which was available in both paper-and-pencil and electronic versions. The introduction letters and questionnaires were distributed digitally by both postal mail and the secure electronical mail service, Digital Post. The questionnaire included 98 questions, of which approximately 50% had underlying items. The data collection procedure is described in detail elsewhere [33]. The Building and Housing Register was used to obtain information on type of housing [35].

In all, 14,022 individuals answered the questionnaire (corresponding to 56% of the invited sample), out of which 3893 individuals lived in multi-storey housing. Unit non-response was associated with male sex, younger age, being unmarried and a non-Danish ethnic background [33].

In the questionnaire, noise annoyance was assessed by asking the respondent whether they had been annoyed by noise from neighbours (inside their home) during the past two weeks. The response options were threefold: ‘Yes, very annoyed’, ‘Yes, slightly annoyed’, and ‘No’. In all, 3509 of the respondents answered this question. The prevalence of bothering health symptoms was assessed by asking the respondent whether he or she had been bothered by eight different health symptoms covering both physical and mental outcomes during the past two weeks (in the same order as presented here): ‘Pain or discomfort in the shoulder or neck’, ‘Pain or discomfort in the arms, hands, legs, knees, hips, or joints’, ‘Pain or discomfort in the back or lower back’, ‘Fatigue’, ‘Headache’, ‘Sleeping problems or insomnia’, ‘Melancholy, depression or unhappiness’, and ‘Anxiety, nervousness, restlessness or apprehension’. There were three response options for each symptom: ‘Yes, very bothered’, ‘Yes, slightly bothered’, and ‘No’. These questions on noise annoyance and bothering health symptoms, respectively, were not placed at the same location within the questionnaire. Hence, the question on noise annoyance were placed at the end of the questionnaire, while the questions on bothering health symptoms was placed at the beginning of the questionnaire.

The variables included as possible confounding factors (age, sex, marital status, degree of urbanisation, highest level of completed education and ethnic background) were selected a priori based on our knowledge of the previous literature [e.g. 8, 10, 27, 28]. Furthermore, owner/tenant status was considered a possible confounder and was used as a dichotomous variable. Information on owner/tenant status was obtained from the Building and Housing Register [35]. Information on age, sex, marital status and ethnic background was obtained from the Danish Civil Registration System. Ethnic background was classified according to information on the respondent’s citizenship, country of birth and parental country of birth, and were divided into three groups [36]. Information on the highest completed level of education was based on self-reported information from the questionnaire and categorised as: ‘Basic school’, ‘Upper secondary or vocational education’, ‘Higher education’ or ‘Other or in school’. Education is a stable indicator over time (i.e. changing little during adulthood) and is strong predictor of social class and income [37]. The Danish municipalities were grouped into three types of areas according to Eurostat’s classification of urban and rural areas [38].

The questionnaire also included other possible confounding factors. For example, the respondents were asked whether they had been annoyed by noise from traffic (inside their home) during the past two weeks. The possible response options were: ‘Yes, very annoyed’, ‘Yes, slightly annoyed’, and ‘No’. Furthermore, the questionnaire included a chronic condition checklist. The following question was asked: ‘For each of the following conditions and health problems, please indicate whether you have it now or have had it earlier. If you’ve had it before, please also indicate whether you have after-effects’. The following health conditions were included in the present study: diabetes, cancer, myocardial infarction or angina pectoris and chronic obstructive pulmonary disease. Respondents who answered affirmatively to currently having a specific health condition or who reported having after-effects of the specific health condition were classified as having the health condition of interest.

### Statistical methods

The prevalence of experiencing bothering health symptoms according to the degree of the symptom is presented as percentages with 95% confidence intervals (CIs). The confidence intervals were calculated using the Wilson Score method. Multiple logistic regression models were used to investigate the associations between neighbour noise annoyance and each of the health symptoms. Hence, each of the eight outcome variables were dichotomized into ‘Very bothering health symptoms’ versus ‘Slightly bothering health symptoms’ or ‘No’. The results of the logistic regression models are reported as odds ratios (ORs) with 95% CIs. The ORs are adjusted for age, sex, marital status, degree of urbanisation, owner/tenant status, highest level of completed education and ethnic background. We also sought to investigate the potential interaction between sex and neighbour noise annoyance in relation to the eight different health symptoms. Statistically significant interactions between sex and neighbour noise annoyance were observed for two outcome indicators: ‘Pain or discomfort in the shoulder or neck’ and ‘Pain or discomfort in the arms, hands, legs, knees, hips or joints’. Thus, these two regression models were stratified by sex. In an additional model, we further adjusted for traffic noise annoyance during the past two weeks and self-reported morbidity (i.e. diabetes, cancer, myocardial infarction or angina pectoris and chronic obstructive pulmonary disease). A calibration weighting technique was used to reduce non-response bias [33, 39]. SAS version 9.4 was used for all analyses. The survey was approved by the Danish Data Protection Agency.

## Results

Table 1 presents the characteristics of the study population (*n* = 3509). According to the table, a total of 6.7% of the respondents living in multi-storey housing in 2017 reported being very annoyed by neighbour noise during the past two weeks, whereas 28.9% had been slightly annoyed.

**Table 1:** Characteristics of the study population (individuals living in multi-storey housing in 2017). Percentages

		Annoyed by noise from neighbours				
		Yes, very annoyed	Yes, slightly annoyed	No	Total	No. of respondents
All		6.7	28.9	64.4	100.0	3509
Sex						
Men		5.9	28.8	65.4	100.0	1524
Women		7.5	29.1	63.5	100.0	1985
Age						
16–24 y.		7.6	34.9	57.5	100.0	534
25–44 y.		8.5	33.6	57.9	100.0	1208
45–64 y.		6.4	27.7	65.9	100.0	905
≥ 65 y.		2.9	16.6	80.5	100.0	862
Cohabitation status						
Married or cohabiting		6.0	28.0	66.0	100.0	1779
Not married or cohabiting		7.3	29.8	62.9	100.0	1730
Education						
Basic school		5.8	25.6	68.7	100.0	224
Upper secondary or vocational school		8.1	29.6	62.3	100.0	1024
Higher education		5.9	29.0	65.1	100.0	1560
Other		7.2	31.5	61.3	100.0	588
Degree of urbanisation						
Densely populated area		6.7	30.4	62.8	100.0	2337
Intermediate		5.4	25.5	69.2	100.0	507
Thinly populated area		7.5	26.5	66.0	100.0	665
Country of origin						
Denmark		6.6	29.6	63.8	100.0	3047
Other western		7.7	27.0	65.3	100.0	178
Non-western		6.5	26.0	67.5	100.0	284
Owner/tenant status						
Owner		3.4	23.5	73.1	100.0	611
Tenant		7.1	29.9	63.0	100.0	2561

Neighbour noise annoyance within the past two weeks was strongly associated with all included health symptoms (all *P*-values < 0.05) (Table 2). For example, the prevalence of being very bothered by fatigue during the past two weeks decreased from 33.0% among those highly annoyed by noise from neighbours to 20.4 and 13.9% among those slightly and not at all annoyed, respectively.

**Table 2:** Prevalence of bothering health symptoms within the past two weeks by noise annoyance from neighbours within the past two weeks (among individuals living in multi-storey housing in 2017). Percentages

		Annoyed by noise from neighbours			
		Yes, very annoyed	Yes, slightly annoyed	No	P-value
Pain or discomfort in the shoulder or neck	Yes, very bothered	24.1	16.7	13.8	< 0.0001
	Yes, slightly bothered	38.4	41.6	34.4	
	No	37.5	41.7	51.8	
	Total	100.0	100.0	100.0	
Pain or discomfort in the arms, hands, legs, knees, hips or joints	Yes, very bothered	24.4	15.4	15.1	0.0194
	Yes, slightly bothered	32.1	36.9	35.0	
	No	43.5	47.7	49.9	
	Total	100.0	100.0	100.0	
Pain or discomfort in the back or lower back	Yes, very bothered	25.6	15.6	14.5	< 0.0001
	Yes, slightly bothered	37.3	40.6	34.1	
	No	37.1	43.8	51.4	
	Total	100.0	100.0	100.0	
Fatigue	Yes, very bothered	33.0	20.4	13.9	< 0.0001
	Yes, slightly bothered	45.5	53.7	45.3	
	No	21.5	25.9	40.8	
	Total	100.0	100.0	100.0	
Headache	Yes, very bothered	15.5	8.9	7.0	< 0.0001
	Yes, slightly bothered	35.0	37.0	26.4	
	No	49.5	54.1	66.6	
	Total	100.0	100.0	100.0	
Sleeping problems or insomnia	Yes, very bothered	24.5	15.5	10.9	< 0.0001
	Yes, slightly bothered	39.3	36.6	27.2	
	No	36.2	47.9	61.9	
	Total	100.0	100.0	100.0	
Melancholy, depression or unhappiness	Yes, very bothered	16.4	9.7	7.2	< 0.0001
	Yes, slightly bothered	34.7	31.1	24.6	
	No	48.9	59.2	68.1	
	Total	100.0	100.0	100.0	
Anxiety, nervousness, restlessness or apprehension	Yes, very bothered	16.4	10.1	6.9	< 0.0001
	Yes, slightly bothered	37.7	31.7	23.3	
	No	45.9	58.2	69.8	
	Total	100.0	100.0	100.0	

Table 3 shows the results of the multiple logistic regression analyses, and as can be seen there are strong associations between neighbour noise annoyance and being very bothered by all health symptoms in a dose-dependent manner, even after adjustment for potential confounding factors (all *P*-values < 0.05). For example, individuals who had been very annoyed by noise from neighbours during the past two weeks had 2.91 (95% CI: 2.14–3.98) times higher odds of being very bothered by fatigue during the same reference period compared to individuals who had not been annoyed by noise from neighbours. Similar results were observed when the models were further adjusted for traffic noise annoyance and self-reported morbidity (data not shown). Interestingly, after adjustment for these covariates, the odds ratios increased slightly in six out of eight symptoms among individuals who had been very annoyed by noise from neighbours. On the other hand, the odds ratios decreased somewhat for most symptoms among individuals who had been slightly annoyed by noise from neighbours.

**Table 3:** Adjusted odds ratios (and 95% confidence intervals) of very bothering health symptoms within the past two weeks by noise annoyance from neighbours within the past two weeks (among individuals living in multi-storey housing in 2017)

	Annoyed by noise from neighbours					
	Yes, very annoyed		Yes, slightly annoyed		No	P-value
Pain or discomfort in the shoulder or neck	1.73	(1.22–2.45)	1.32	(1.06–1.65)	1	0.0016
Pain or discomfort in the arms, hands, legs, knees, hips or joints	2.23	(1.57–3.17)	1.29	(1.03–1.61)	1	< 0.0001
Pain or discomfort in the back or lower back	3.32	(2.15–5.13)	1.57	(1.15–2.14)	1	< 0.0001
Fatigue	2.91	(2.14–3.98)	1.46	(1.18–1.79)	1	< 0.0001
Headache	1.82	(1.19–2.78)	1.16	(0.87–1.54)	1	0.0221
Sleeping problems or insomnia	2.62	(1.86–3.69)	1.46	(1.16–1.84)	1	< 0.0001
Melancholy, depression or unhappiness	2.10	(1.39–3.18)	1.46	(1.11–1.92)	1	0.0004
Anxiety, nervousness, restlessness or apprehension	2.60	(1.73–3.91)	1.58	(1.20–2.09)	1	< 0.0001

^1^Adjusted for sex, age, marital status, education, degree of urbanisation, owner/tenant status and ethnic background

**Fig. 1:**
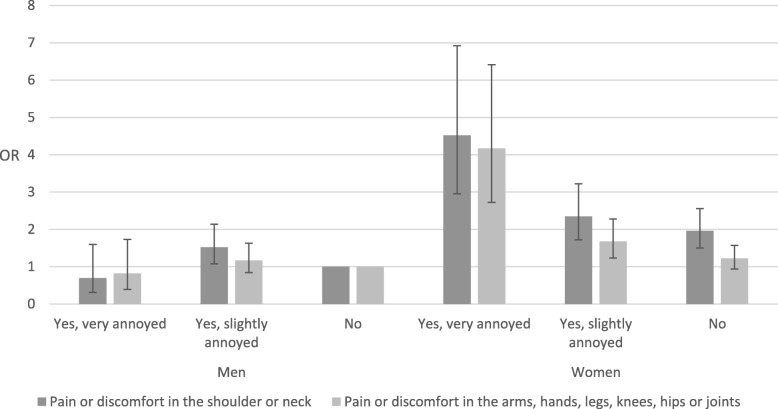
Sex-stratified odds ratios (OR) and 95% confidence intervals for having pain or discomfort in the shoulder or neck, or in the arms, hands, legs, knees, hips or joints within the past two weeks, respectively, with noise annoyance from neighbours within the past two weeks (among individuals living in multi-storey housing in 2017)

As previously stated, our analyses revealed sex-specific interactions for the association between neighbour noise annoyance and being very bothered by pain or discomfort in the shoulder or neck, and pain or discomfort in the arms, hands, legs, knees, hips or joints, respectively, within the past two weeks. Thus, sex-stratified analyses were carried out for these two outcome measures, which showed that women who reported being highly annoyed by noise from neighbours had 4.52 (95% CI: 2.95–6.92) times higher odds of having very bothering pain or discomfort in the shoulder or neck, and 4.17 (95% CI: 2.72–6.41) times higher odds of having very bothering pain or discomfort in arms, hands, legs, knees, hips or joints (Fig. 1). The reference group was men who had not been annoyed by noise from neighbours. Associations were also significant for women who had been slightly annoyed by noise from neighbours although by a smaller magnitude. No associations were found for men for the two health symptoms of pain or discomfort.

## Discussion

Based on previous research suggesting noise annoyance to be adversely linked to indicators of both physical and mental health, we investigated the possible associations between neighbour noise annoyance and a total of eight different physical and mental health symptoms in a nationwide sample of adults in Denmark living in multi-storey housing. Our results revealed a clear relationship between neighbour noise annoyance and all included health symptoms. Moreover, sex-specific associations were demonstrated for two indicators (i.e. ‘Pain or discomfort in the back/neck’ and ‘Pain or discomfort in the arms/hands/legs/knees/hips/joints’), where only significant associations were found for women.

Sex-specific effects of noise indicators have been documented in only a few previous studies and with somewhat conflicting results [e.g. 29–32]. For example, Heinonen-Guzejev and colleagues found self-reported noise-sensitivity to increase cardiovascular mortality among women, but not among men [29]. However, the question on noise sensitivity in this study more likely reflected noise annoyance, as the respondents were asked whether they were disturbed by noise. Thus, the question resembled the one on noise annoyance in our study to a great extent. Moreover, in a large hospital-based case-control study carried out in Berlin, associations were found between traffic noise exposure and the risk of myocardial infarction in men, but not in women [30], and between diurnal noise annoyance and the risk of myocardial infarction in women, but not in men [31]. Also, in a study by Nivison and Endresen, cardiovascular complaints were related to noise sensitivity in women, but not in men [32]. Based on these findings and albeit apparently slightly conflicting results, it seems as if sex-specific effects of noise on health may depend on whether the noise exposure could be quantified in terms of physical parameters or by the level of noise annoyance/noise sensitivity, with men being more susceptible to the former and women to the latter.

One possible explanation of such sex-specific effects of noise on health may be that women spend more time at home and therefore are more likely to experience an increased noise exposure [31]. According to official registry statistics in Denmark [40], the employment status distribution is almost equal between men (53%) and women (47%) and is therefore unlikely to fully explain the difference between men and women demonstrated in the present study. However, a nationally representative Danish survey shows that the average time spent on paid work, housework and leisure activities, respectively, varies between men and women e.g. with men working more hours per week than women, but with women spending more time doing housework during the week and taking care of children than men [41]. This means that it is likely that women are at home more than men. Thus, based on the tendencies documented in this report, differences between sexes in the time spent at home is likely to at least partly explain the sex-specific association between noise annoyance and symptoms of physical pain, simply because of a higher potential exposure to noise from neighbours among women.

Another possible explanation for the demonstrated sex-specific associations between noise annoyance and physical health symptoms of pain may be related to the complex nature and origin of noise sensitivity/annoyance. Susceptibility to negative reactions to noise could be expanded to other areas of sensitivity among affected individuals [21]. Therefore, a more general sensitivity could also plausibly apply to reactions to other stimuli related to e.g. health conditions, including physical symptoms of pain. In their hypothesis of negative affectivity, Watson and Clark characterised negative affectivity as a stable and pervasive personality dimension characterised by individuals who are more inclined to report distress, discomfort, and dissatisfaction, even in the absence of obvious stressors [42]. Moreover, it is well known that even though women live longer than men, women generally report worse health (e.g. in relation to self-reported health, mental health, sleeping problems, pain and discomfort) than men [43–45]. This contrast is known as the male-female health survival paradox. Bonke and Christensen stratified their analyses according to average weekly working hours and found a larger proportion of women than men experience high levels of perceived stress within the same strata [41]. Thus, it is likely that women are more neurobiologically sensitive, which is also reflected by a stronger association between noise sensitivity and physical symptoms of pain. However, we did not find sex-specific associations for all included eight health symptoms, which suggests that a sex-dependent neurobiological sensitivity may not apply to all types of symptoms.

Overall, our results on the association between neighbour noise annoyance and physical and mental health symptoms are in line with previous research, indicating an adverse impact on a broad range of physical and mental health symptoms [7, 10, 13, 26, 27]. Comparisons to other studies are generally compromised, however, as different measures of both noise annoyance and indicators of physical and mental health are used.

As mentioned initially, a suggested model that may biologically explain the association between noise annoyance and health is related to the physical stress-response that can be triggered in some individuals as a response to environmental noise exposure. Münzel and colleagues argue that the model describing the generalized psychophysiological reactions to stress originally formulated in 1977 by Henry and Stephens can also be applied to noise [12]. Applying this model to stress caused by noise annoyance, Meyer and Wirtz argue in their review that in the case of chronic stress response stimulation, dysfunctions in the hypothalamic-pituitary-adrenal (HPA) axis and/or cytokine levels occur [46]. Such dysfunctions may result in several different physical and mental health symptoms, as the HPA axis activity and cytokine levels regulate several conditions such as immune-modulatory effects, mood disorders, and sleep fragmentation [47]. Thus, in relation to the results in our study, one might speculate that noise annoyance induces a chronic stress response stimulation, eventually resulting in both physical and mental health symptoms. It should, however, be mentioned that the proposed mechanisms potentially explaining the demonstrated associations between noise annoyance and mental and physical health symptoms should be interpreted with caution, until future studies have thoroughly confirmed such biological mechanisms.

Some limitations in the present study should be noted. As the study is based on cross-sectional data, it is not possible to determine the direction of causality i.e. whether neighbour noise annoyance increases the risk of adverse physical and mental health outcomes, or whether individuals experiencing various health symptoms are more likely to be annoyed by noise from neighbours. While the former points towards a health-damaging impact of noise annoyance on human health, the latter may reflect a general systemic sensitivity or vulnerability towards various stimuli. It should also be noted that respondents were less likely to live in multi-storey housing than in the entire target population. The main reason for this is, with all certainty, a slightly lower response rate among individuals living in multi-storey housing than among individuals living in other types of housing. However, studies of associations are generally less sensitive to non-response bias than prevalence studies and there is no reason to suspect that non-response bias is a major issue in the present study. The use of self-reported data is, of course, a possible limitation, as such data solely relies on the accurate recall of the respondent. However, as noise annoyance is defined as a subjectively experienced phenomenon, as is also the case with the experience of bothering physical and mental health symptoms, self-reports on these indicators may not hamper the validity of data after all. A final limitation of the present study is that noise-induced annoyance was not assessed according to internationally standardised specifications related to noise questions and response scales in social surveys, such as the guidelines in ISO/TS 15666:2003 [48].

A strength of the present study is that the sample is based on a representative random sample of adults aged 16 years or older, which allows us to generalise the findings to the entire adult population living in multi-storey housing in Denmark. Moreover, we believe that restricting our analyses to only those living in multi-storey housing is a key strength of the study, as the study population then is relatively homogenous in relation to housing conditions i.e. how close they live to their neighbours. This means that the associations between neighbour noise annoyance and physical and mental health symptoms of pain and discomfort are not likely to be confounded by a substantial variation in the type of housing among the respondents. To our knowledge, no previous study has restricted analyses on the association between neighbour noise annoyance and various physical and mental health symptoms to only include individuals living in multi-storey housing. Thus, former studies may have underestimated the impact of neighbour noise annoyance on health. Further, the same reference period (two weeks) was used to assess both exposure (i.e. neighbour noise annoyance) and outcome (bothering physical and mental health) symptoms, which strengthens the validity of the demonstrated associations.

## Conclusion

In all, 6.7% of adult Danes living in multi-storey housing reported being very annoyed by neighbour noise during the past two weeks, whereas 28.9% had been slightly annoyed. The results from the present study suggest neighbour noise annoyance to be significantly associated with eight different physical and mental health symptoms such as pain in various body parts, headache, fatigue, depression and anxiety. Sex differences were observed for being very bothered by two health symptoms: ‘Pain or discomfort in the neck or shoulder’ and ‘Pain or discomfort in the arms, hands, legs, knees, hips or joints’. Thus, sex-stratified analyses revealed significant associations with neighbour noise annoyance for women in a dose-dependent manner, but no association was observed for men.

Future studies are encouraged to 1) determine the direction of causality using a longitudinal design and 2) explore in detail the biological mechanisms explaining the sex-specific impact of neighbour noise annoyance on symptoms of musculoskeletal pain or discomfort and the other health symptoms as well.

## Data Availability

The datasets generated and/or analysed during the current study are not publicly available due to regulations formulated by the Danish Data Protection Law and Statistics Denmark (data are located on a secure server at Statistics Denmark). Access to data can only be granted to researchers in Danish research environments after approval from the Danish Data Protection Agency and Statistics Denmark, respectively.

## References

[1] European Environment Agency. Noise in Europe 2014. EEA Report No. 10/2014. Luxembourg: Publications Office of the European Union; 2014.

[2] The Department for Environment, Food and Rural Affairs. National Noise Attitude Survey 2012 (NNAS2012) - Summary Report. The Department for Environment, Food and Rural Affairs. London: UK Government; 2014.

[3] The Department for Environment, Food and Rural Affairs. NANR322 Survey of Noise Attitudes (SoNA) 2013 –NO0242. Report Ref: 47067932.NN1501.R1/02. Nottingham: AECOM Infrastructure & Environment UK Limited, 2015.

[4] World Health Organization (2018). Environmental noise guidelines for the European region.

[5] World Health Organization. Burden of disease from environmental noise. Quantification of healthy life years lost in Europe. Copenhagen: World Health Organization; 2011.

[6] Okokon EO, Turunen AW, Ung-Lanki S, Vartiainen AK, Tiittanen P, Lanki T (2015). Road-traffic noise: annoyance, risk perception, and noise sensitivity in the Finnish adult population. Int J Env Res Public Health..

[7] Beutel Manfred E., Jünger Claus, Klein Eva M., Wild Philipp, Lackner Karl, Blettner Maria, Binder Harald, Michal Matthias, Wiltink Jörg, Brähler Elmar, Münzel Thomas (2016). Noise Annoyance Is Associated with Depression and Anxiety in the General Population- The Contribution of Aircraft Noise. PLOS ONE.

[8] Maschke C, Niemann H (2007). Health effects of annoyance induced by neighbour noise. Noise Control Eng J.

[9] Grimwood CJ. Effects of environmental noise on people at home. Information paper 22/93. UK: BRE; 1993.

[10] Nitschke M, Tucker G, Simon DL, Hansen AL, Pisaniello DL (2014). The link between noise perception and quality of life in South Australia. Noise Health..

[11] van Kempen E, Babisch W (2012). The quantitative relationship between road traffic noise and hypertension: a meta-analysis. J Hypertens.

[12] Münzel T, Gori T, Babisch W, Basner M (2014). Cardiovascular effects of environmental noise exposure. Eur Heart J.

[13] Niemann H, Bonnefoy X, Braubach M, Hecht K, Maschke C, Rodrigues C, Röbbel N (2006). Noise-induced annoyance and morbidity results from the pan-European LARES study. Noise Health.

[14] Eze IC, Foraster M, Schaffner E, Vienneau D, Héritier H, Pieren R (2018). Transportation noise exposure, noise annoyance and respiratory health in adults: a repeated-measures study. Environ Int.

[15] Zare Sakhvidi MJ, Zare Sakhvidi F, Mehrparvar AH, Foraster M, Dadvand P (2018). Association between noise exposure and diabetes: a systematic review and meta-analysis. Environ Res.

[16] Guski R, Schreckenberg D, Schuemer R (2017). WHO environmental noise guidelines for the European region: a systematic review on environmental noise and annoyance. Int J Environ Res Public Health.

[17] Sung Joo, Lee Jiho, Jeong Kyoung, Lee Soogab, Lee Changmyung, Jo Min-Woo, Sim Chang (2017). Influence of Transportation Noise and Noise Sensitivity on Annoyance: A Cross-Sectional Study in South Korea. International Journal of Environmental Research and Public Health.

[18] Park J, Chung S, Lee J, Sung JH, Cho SW, Sim CS (2017). Noise sensitivity, rather than noise level, predicts the non-auditory effects of noise in community samples: a population-based survey. BMC Public Health.

[19] Marks A, Griefahn B (2007). Associations between noise sensitivity and sleep, subjectively evaluated sleep quality, annoyance, and performance after exposure to nocturnal traffic noise. Noise Health..

[20] Michaud DS, Feder K, Keith SE, Voicescu SA, Marro L, Than J (2016). Self-reported and measured stress related responses associated with exposure to wind turbine noise. J Acoust Soc Am.

[21] Stansfeld SA. Noise, noise sensitivity and psychiatric disorder: epidemiological and psychophysiological studies. Psychol Med Monogr Suppl. 1992:22.1–44. 1343357

[22] Bullen RB, Hede AJ, Kyriacos E (1986). Reaction to aircraft noise in residential areas around Australian airports. J Sound Vib.

[23] Kjellberg A, Landström U, Tesarz M, Söderberg L, Akerlund E (1996). The effects of non-physical noise characteristics, ongoing task and noise sensitivity on annoyance and distraction due to noise at work. J Environ Psychol.

[24] van Kamp I, Job RF, Hatfield J, Haines M, Stellato RK, Stansfeld SA (2004). The role of noise sensitivity in the noise-response relation: a comparison of three international airport studies. J Acoust Soc Am..

[25] European Parliament (2002). Directive 2002/49/EC of the European Parliament and of the council of 25^th^ June 2002 relating to the assessment and management of environmental noise: official journal of the European Communities, L189.

[26] Shepherd D, Welch D, Dirks KN, McBride D (2013). Do quiet areas afford greater health-related quality of life than noisy areas?. Int J Environ Res Public Health.

[27] Hammersen F, Niemann H, Hoebel J (2016). Environmental noise annoyance and mental health in adults: findings from the cross-sectional German health update (GEDA) study 2012. Int J Env Res Public Health.

[28] Jensen HAR, Rasmussen B, Ekholm O (2018). Neighbour and traffic noise annoyance: a nationwide study of associated mental health and perceived stress. Eur J Pub Health.

[29] Heinonen-Guzejev M, Vuorinen HS, Mussalo-Rauhamaa H, Heikkilä K, Koskenvuo M, Kaprio J (2007). The association of noise sensitivity with coronary heart and cardiovascular mortality among Finnish adults. Sci Total Environ.

[30] Babisch W, Beule B, Schust M, Kersten N, Ising H (2005). Traffic noise and risk of myocardial infarction. Epidemiology..

[31] Willich SN, Wegscheider K, Stallmann M, Keil T (2006). Noise burden and the risk of myocardial infarction. Eur Heart J.

[32] Nivison ME, Endresen IM (1993). An analysis of relationship among environmental noise, annoyance and sensitivity to noise, and the consequences for health and sleep. J Behav Med.

[33] Jensen HAR, Ekholm O, Davidsen M, Christensen AI (2019). The Danish health and morbidity surveys: study design and participant characteristics. BMC Med Res Methodol.

[34] Pedersen CB (2011). The Danish civil registration system. Scand J Public Health..

[35] Christensen G (2011). The building and housing register. Scand J Public Health.

[36] Kurita GP, Sjøgren P, Juel K, Højsted J, Ekholm O (2012). The burden of chronic pain: a cross-sectional survey focussing on diseases, immigration, and opioid use. Pain..

[37] d’Errico A, Ricceri F, Stringhini S, Carmeli C, Kivimaki M, Bartley M (2017). Socioeconomic indicators in epidemiologic research: a practical example from the LIFEPATH study. PLoS One.

[38] Eurostat. Urban-rural typology. Eurostat. Statistics explained. 2017. Available at: https://ec.europa.eu/eurostat/statistics-explained/index.php/Archive:Urban-rural_typology (19 March 2019, date last accessed).

[39] Särndal C-E, Lundström S (2005). Estimation in surveys with non-response.

[40] Denmark S (2017). Register-based labour force, employment.

[41] Bonke J, Christensen AEW (2018). How do the Danes spend the time? [in Danish].

[42] Watson D, Clark LA (1984). Negative affectivity: the disposition to experience aversive emotional states. Psychol Bull.

[43] Oksuzyan A, Gumà J, Doblhammer G, Doblhammer G, Gumà J (2018). Sex differences in health and survival. A demographic perspective on gender, family and health in Europe.

[44] Dahlin J, Härkönen J (2013). Cross-national differences in the gender gap in subjective health in Europe: does country-level gender equality matter?. Soc Sci Med.

[45] National Institute of Public Health & the Danish Health Authority. Health in Denmark – Results from the Danish National Health Survey. 2018. http://www.danskernessundhed.dk/. Accessed 5 Jul 2019.

[46] Meyer T, Wirtz PH (2018). Mechanisms of mitochondrial redox signalling in psychosocial stress-responsive systems: new insights into an old story. Antioxid Redox Signal.

[47] Daiber A, Kröller-Schön S, Frenis K, Oelze M, Kalinovic S, Vujacic-Mirski K (2019). Environmental noise induces the release of stress hormones and inflammatory signalling molecules leading to oxidative stress and vascular dysfunction – signatures of the internal exposome. Biofactors..

[48] ISO/TS 15666. Acoustics — Assessment of noise annoyance by means of social and socio-acoustic surveys. Genève, ISO; 2003.

